# ≤ Cyclin D1 protein affecting global women’s health by regulating HPV mediated adenocarcinoma of the uterine cervix

**DOI:** 10.1038/s41598-019-41394-9

**Published:** 2019-03-22

**Authors:** Richa Tripathi, Gayatri Rath, Poonam Jawanjal, Mausumi Bharadwaj, Ravi Mehrotra

**Affiliations:** 1 Division of Molecular Genetics & Biochemistry, ICMR-National Institute of Cancer Prevention and Research (NICPR), Noida, India; 2 Division of Preventive Oncology, ICMR-National Institute of Cancer Prevention and Research (NICPR), Noida, India; 3 Department of Anatomy, VMMC & Safdarjung Hospital, New Delhi, India

## Abstract

Adenocarcinoma (ADC) of the uterine cervix (UC) is a rare form of cervical cancer (CC) caused due to the infection of Human Papilloma Virus (HPV). Cyclin D1 is one of the downstream targets of aberrantly activated Notch signaling, contribute to the etiology of CC. However, little is known about the role of Cyclin D1 in the modulation of cervical ADC and is controversial. The purpose of this study is to determine the role of Cyclin D1 protein and to elucidate the combined analysis with Notch signaling proteins in HPV associated ADCs of CC. A total of 60 biopsy samples (40 normal and 20 ADCs of CC) were analyzed for the expression of Cyclin D1 in HPV associated ADCs via immunohistochemistry and by immunoblotting. HPV-16 positive ADC patients showed a strong association with the Cyclin D1 expression (p = 0.007). The significant mean difference (p = 0.0001) and the pairwise comparison between Cyclin D1/JAG1 (p = 0.0001), and Cyclin D1/Notch-3 (p = 0.0001) were observed. The above Notch signaling proteins showed their synergistic role in modulating Cyclin D1 which in-turn regulates HPV-16 associated ADC of the uterine cervix (UC), affecting women’s global health.

## Introduction

Cervical cancer (CC), with an estimated incidence rate 528,000 annually has been emerged as the fourth most leading women related cancer worldwide^1^. Hence, newer diagnostic methodologies have led to improve early cancer diagnosis of CC, leading to minimize morbidity and better disease prognosis. However, CC is detected in 80% of advanced cancer stage in  India with 453.02 million women population are at risk of developing cervical cancer and contributes more than 1/5^th^ of global burden due to inappropriate screening. It ranked second among women affecting cancers with age group of 15–44 years with its alarming effect due to social and economic cost^1^.

Harold zur Hausen, a German virologist showed first time the association between genital HPV infections and cervical cancer in the early 1980s. High-risk human papillomavirus (HRHPV)-16/18, the major etiological factor and the overexpression of HPV oncogenes E6 and E7 are reported to be linked in malignant transformation and the development of CC^2^. However, HPV infections are not found to be solely associated with the development of CC as many of them are transient^3^. Therefore, the other factors including deregulated interconnected signaling pathways like Notch, WNT/β-catenin have also been associated with HPV infected CC patients^4^. Besides above, the altered molecular mechanisms for pathway activation modulating their effector target proteins leading to CC remain unknown.

CC is classified into invasive squamous cell carcinoma (ISCC, 85 to 90% in India) and Adenocarcinoma (ADC, 10–15%)^5^. ADC of uterine cervix (UC) is a rare form of cervical cancer (CC) which develops from mucinous endocervical epithelium^5,6^. Persistent HPV infection result in the development of invasive ADC. Although, the prevalence of ISCC is significantly decreased^1^ but for ADC it is reported to be distressingly increased worldwide^6^. In North Indian hospitals, ADC patients of CC are rare and therefore, difficult to be included in the study. Majority of ADC patients report to the hospitals at late stages and therefore, diagnosis occurs at late stage, and hence, accounted with worse prognosis outcome as there is no effective systemic therapy. In addition, there is no targeted therapy for ADC of uterine cervix due to poor understanding of the molecular mechanisms behind the development and progression of ADC, strengthening the need for its early diagnosis and treatment via targeted therapies.

Notch signaling pathway plays an important role in cancer cell proliferation, differentiation, cell fate specification, maintaining multicellular homeostastsis, vasculogenesis, angiogenesis, epithelial-mesenchymal transition (EMT), and metastasis^7^. However, the complete mechanism is poorly understood. Five ligands including (three Delta-like and two Jagged/Serrate) and four Notch homologues (Notch 1–4) were reported to be associated with Notch signaling^8^. Previously, our laboratory showed upregulation of JAG1 and Notch-3 leading to the activation of JAG1 induced Notch-3 signaling pathway in cervical cancer^9,10^. This activation of Notch signaling leads to translocation of cleaved intracellular Notch C-terminal fragment to nucleus resulting in accumulation of CSL(CBF1, Suppressor of Hairless, Lag1), Mastermind-like proteins (MAML1, 2 and 3) and p300 proteins and alters the transcription of various downstream target genes^11^. Recently, we have shown the role of upregulated HES1 (Hairy and enhancer of split homolog-1) protein, downstream effector target of mammalian Notch signaling in HPV-16 associated ADCs of CC^12^.

Cyclin D1 is also one of the important target gene of Notch signaling pathway and is one of the family members of the D-type cyclins which regulates the G1/S phase transition of cell cycle and is reported to be associated with tumor biology of human cells^13^. Till date, little and controversial data is available^14–19^ about the expression of Cyclin D1 in invasive ADC. Continuing our focus on Notch signaling, this study aims to study the expression of Cyclin D1 protein along with association among Cyclin D1 and Notch signaling proteins- Notch-3 and Jagged-1. In addition, this study determines the biological as well as clinical relevance of Cyclin D1 in the development and the progression of HPV associated invasive ADCs of UC.

## Results

### Cyclin D1 and HPV infected ADC cases

The association of Cyclin D1 with HPV-16 positive ADC patients was observed to be significant (p = 0.007, Table 1). We could not do the comparison of HPV-18 positive/negative ADC patients with the expression of Cyclin D1 as only one patient was observed to be infected with HPV-18.

**Table 1:** Correlation of Cyclin D1 expression in HPV infected ADC cases.

HPV type	Status	ADC cases (n = 20)	CyclinD1 nuclear		p-value
			−n (%)	+n (%)	
HPV-16	No	03	02 (66.6)	01 (33.3)	**0.007**
HPV-16	Yes	17	01 (5.9)	16 (94.1)	
HPV-18	No	19	03 (15.8)	16 (84.2)	0.666
HPV-18	Yes	01	0 (0)	01 (100)	

Abbreviations: ADC, Adenocarcinoma; n, number of subjects.

Cyclin D1 protein regulates cell cycle in the nucleus whose increased expression levels in the nucleus confirms it’s regulation by Notch signaling pathway via synergizing with HPV-16 in ADC.

### Combined impact of Notch signaling proteins on ADC

**Figure 1:**
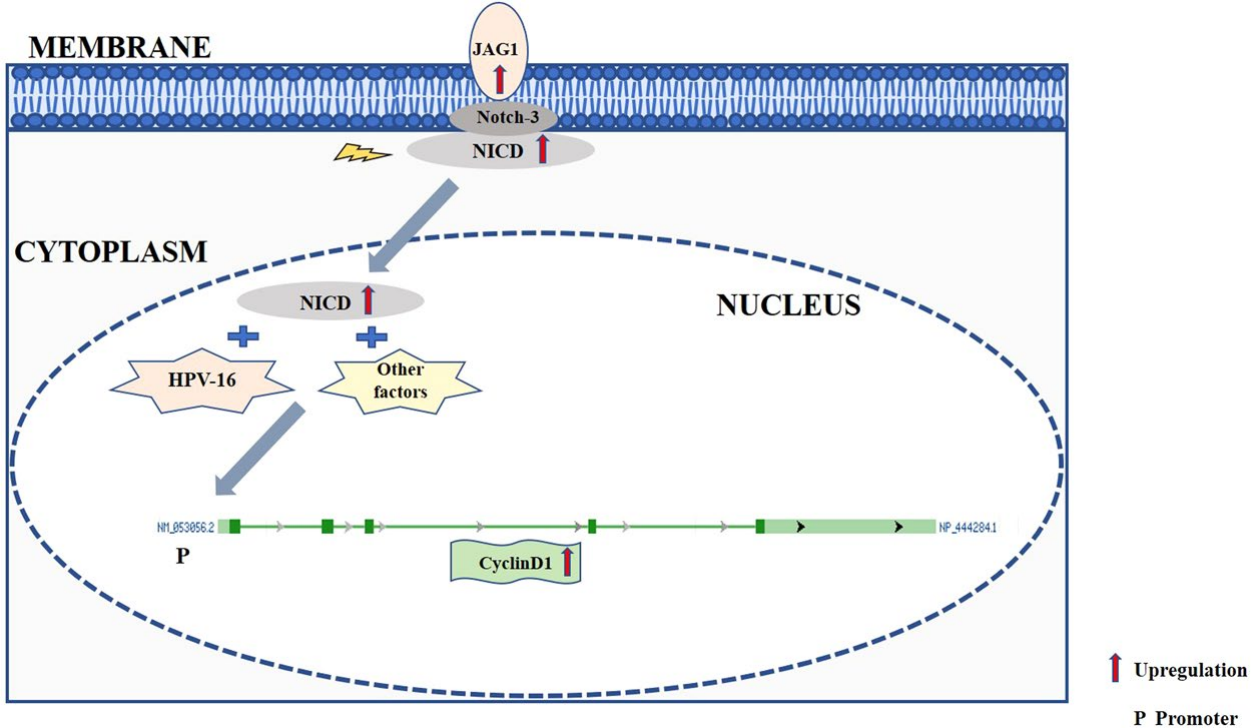
Overview of Cyclin D1 regulation in ADC of uterine cervix. Notch-3 and JAG1 along with HPV-16 signals activates Notch signaling pathway by binding of Notch-3 with JAG1. The NICD domain of Notch-3 further translocates to nucleus and along with signals of HPV-16 resulted in upregulated transcription and translation of Cyclin D1 in the nucleus which regulates cell cycle in ADC of uterine cervix. NICD, Notch Intracellular domain.

The combined effect of Notch signaling proteins- Cyclin D1, JAG1 and Notch-3 on ADC was analyzed for which the JAG1 data of ADC from our recent publication^9^ and Notch-3 data of ADC from our unpublished records (IHC figures available upon request) was used. Among all three Notch signaling pathway proteins (Cyclin D1, Notch-3 and JAG1) the significant mean difference (p = 0.0001, Table 2) and the pairwise comparison between Cyclin D1 and Notch-3, Cyclin D1 and JAG1 were found to be statistically significant (p = 0.015; p = 0.0001, Table-S2) respectively. The above results suggest the significant upregulation and interlinking of Notch-3, JAG1 and Cyclin D1. Hence, the upregulated Notch-3 binds with upregulated JAG1, leading to the activation of Notch signaling in conjuction with HPV-16 which, in turn, alters the expression of downstream target of Notch Signaling Cyclin-D1 in ADC of UC. Therefore, all the three linked proteins should be studied further to understand the complete molecular cascade regulation ADC (Fig. 1).

**Table 2:** Total expression score (Intensity score + Percentage positivity) of Cyclin D1, JAG1 and Notch-3 expression in Normal and ADC tissues.

Proteins	Normal (n = 40)			ADC (n = 20)					
	Mean	±S.E	SD	Mean	±S.E	S.D	Median	*p-value	**p-value
Cyclin D1	1.02	0.28	1.83	4.70	0.50	2.25	6.0	0.0001	0.0001
JAG1	0.95	0.21	1.35	4.20	0.40	1.80	5.0	0.0001	
Notch-3	0.70	0.20	1.30	1.20	0.49	2.12	6.0	0.0001	

Abbreviations: n, number of subjects; p, pvalue; S.D, standard deviation; S.E, standard error.

^*^The comparison of mean between normal vs. ADC independently in Cyclin D1, JAG1 and Notch-3 via chi-square test.

^**^The overall significant mean difference between Cyclin D1, JAG1 and Notch-3 proteins through Kruscal wallis test.

### Cyclin D1 expression in ADC and Receiver Operating Characteristic analysis

**Figure 2:**
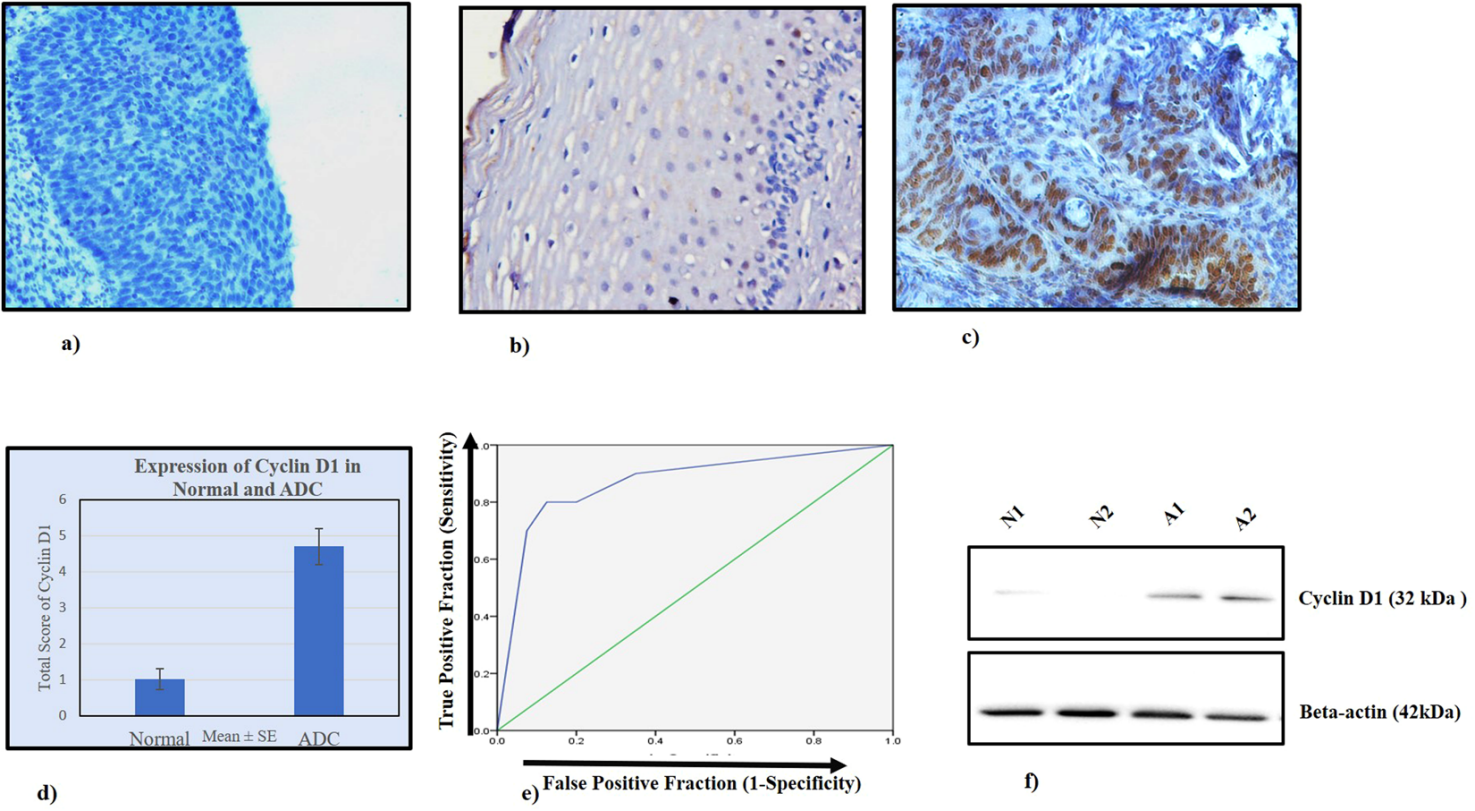
Expression analysis of Cyclin D1 in normal and ADC tissues of uterine cervix. IHC depicting (**a**) negative control in normal tissue, 200X (**b**) Loss of nuclear expression of CyclinD1 in normal cervix, 200X (**c**) intense nuclear localization of Cyclin D1 in ADC, (**d**) Bar graph showing expression of Cyclin D1 in normal and ADC (**e**) Receiver operating characteristic curve of nuclear Cyclin D1 in Normal vs. ADC; Y-axis represents true-positive fraction whereas the X-axis shows false positive fraction. (**f**) Western blots depict the expression of Cyclin D1 in normal (N1, N2) and ADC (A1, A2) of cervical cancer tissues.

The expression profile (Fig. 2a–c; Table 2) of Cyclin D1 in the nucleus was observed in Normal, and ADC tissues. Around four-fold increase of nuclear expression was identified in N (Mean ± S.E, 1.02 ± 0.28) vs. ADC (Mean ± S.E, 4.70 ± 0.50; p = 0.0001). The immunohistochemical total expression scores of Cyclin D1 protein in normal cervix, and ADC biopsies were depicted in Fig. 2d.

## ROC Analysis

### Potential determination of Cyclin D1 expression in order to distinguish ADC from normal cervix tissue

For Cyclin D1, the area-under-the-curve (AUC) values for ADC (0.86; p = 0.0001) along with their sensitivity (80%) and specificity (80%) were observed (Fig. 2e and Table S1).

High sensitivity and specificity of Cyclin D1 in ADC support the clinical utility of Cyclin D1 for progression of ADC.

### Immunoblotting

The findings of immunohistochemistry were validated by immunoblotting. The results of immunoblotting showed the increase in the expression of Cyclin D1 (32 kDa) identified in ADC patient samples (Fig. 2f).

## Discussion

High-risk human papillomavirus (HR-HPV) is currently indicated to be associated in the carcinogenesis of ADC of UC^16^. There is lack of targeted therapy in CC due to poor understanding of signaling pathways underlying CC development and progression. Since, altered signaling pathways are important to understand the complete molecular mechanism affecting CC, therefore, the activation of JAG1 induced Notch signaling via interacting synergistically with HPV oncoproteins E6 and E7 in ISCC and ADC of CC was previously reported from our laboratory^9,10^. Our previous findings on HPV associated ISCCs showed the significant loss of Notch-1 in 18.4% (18/98) and gain of Notch-3 protein in 84.7% (83/98) respectively^10^. In addition, increased expression of JAG1 protein was observed earlier in ISCC as well as ADC cases^9^ confirming the deregulated Notch pathway leading to uncontrolled cell cycle and genetic alterations. This results in triggering of E6 and E7 HPV oncoproteins suggesting the complex interaction between Notch signaling and papillomaviruses strengthening the development of CC. Recently, our laboratory also reported the association of HPV-16 positive ADC with altered HES1(Hairy enhancer split 1), downstream molecule of Notch signaling pathway^12^.

Cyclin D1 expression is found to be an  another downstream target protein of Notch signaling pathway in cervical cancer^11,20–22^ and is suggested to be induced by Notch-ICD domain^23^. However, in diverse cellular situations, a cross-talk between Notch and NF-κB signaling pathways has been reported, affecting the expression of Cyclin D1^24^. Cyclin D1, is an important protein in-charge of regulating cellular cycles from G1 (gap1) to S (synthesis) phase transition in various cell types^17,25^. Cyclin D1 associates along with cyclin-dependent kinase (CDK) 6 or CDK4 at G1 stage which joins with retinoblastoma protein (pRb) for regulating G1/S phase check point in order to induce proliferation of cells^26^. Higher Cyclin D1 levels may reduce time period of G1 phase and increase cell proliferation^27^. Hence, it functions in cellular adhesion, motility, proliferation and stromal invasion leading to carcinogenesis^22,23^. This study has recruited 20 HPV associated invasive ADC of UC patients and 40 normal controls to compare the Cyclin D1 expression in HPV-16/18 negative and positive ADC of UC. Cyclin D1 was found to be significantly associated with HPV-16 infected ADC patients. Significant combined impact of Notch signaling proteins- Cyclin D1, JAG1 and Notch-3 and significant mean difference of ‘Cyclin D1 and JAG1’ and ‘Cyclin D1 and Notch-3’ was observed in ADC. Hence, Cyclin D1, JAG1, and Notch-3 are found to be interrelated which confirms Cyclin D1 as an effector target of JAG1 induced Notch signaling and have potential clinical utility in diagnosing ADC of CC. Hence, previously shown altered Notch pathway, in-turn, modulates Cyclin D1 regulating cell cycle of HPV-16 associated ADC of UC.

Various studies have shown the role of cyclin D1 protein in neoplastic transformation and progression of variety of cancers^28,29^. In ISCC of CC, there is discrepancy in the expression of Cyclin D1 as few authors reported the elevated levels^14,30^ and others reported the decreased levels^14,31^. Similarly, in ADC patients of CC, discrepancy in the expression pattern of Cyclin D1 has been observed. Bae *et al*. reported decreased Cyclin D1 expression in only 3 early stage ADC patients (FIGO stage Ib-IIa) and no invasive ADC was recruited in study^31^. Similarly, Park *et al*.^17^, Schorge *et al*.^32^, Little *et al*.^19^ and Cho *et al*.^14^ found downregulated expression of Cyclin D1 in invasive ADC patients with recruitment of only 8, 32, 19 and 6 ADC patients respectively. Balan *et al*.^15^ and Tong *et al*.^18^ recruited 15 and 6 ADC cases respectively and concluded Cyclin D1 as highly expressed invasion marker. Our results are in concordance with Balan *et al*.^15^ and depicted that the expression of Cyclin D1 in ADC of UC was found to be significantly upregulated as compared to normal cervix tissue. We have extended our work with respect to the expression of Cyclin D1 in HPV-16 associated ADC of UC and have shown the association of Cyclin D1 with Notch signaling pathway proteins. This study has also validated the results of immunohistochemistry by western blotting. Hence, this increase of Cyclin D1 may be associated with its invasive potential^33^ in ADC. In addition, various studies have been elucidated the role of cyclin D1 in cell migration^34^. Cyclin D1 may promote cancer cell migration by regulating Rho/Rho-associated protein kinase signaling and matrix deposition of thrombospondin-1 (TSP-1) an extracellular matrix protein^35^. Our result of four-fold higher cyclin D1 expression in the invasive ADC suggests that the activated Notch signaling leads to the progression, migration and invasion of ADC through the induction of cyclin D1. This may in-turn, alters the progression of G1-S phase of cell cycle causing dysregulated cell cycle or apoptosis however, the complete biological mechanism needs to be explored in future. In addition, ROC curve analysis of Cyclin D1 supports the strong clinical significance of nuclear Cyclin D1 for the detection of ADC patients due to its high sensitivity and specificity.

In conclusion, our results unveiled the HPV-16 linked JAG1 driven Notch signaling affecting the transcription of its downstream effector Cyclin D1 gene and further translation and expression of its Cyclin D1 protein in the nucleus. Hence, in order to improve the diagnostic and treatment modalities of ADC of UC, targeted therapies with more sensitivity and specificity and the combined analysis of Cyclin D1, JAG1 and Notch-3 in ADC are required. This pilot study was carried out in an unusual sub-type of CC i.e., adenocarcinoma to understand the role of altered Cyclin D1 induced by activated HPV-16 associated Notch signaling pathway in cell cycle regulation. Moreover, this study also provides the preliminary evidences highlighting the diagnostic and therapeutic potential of this protein in cervical ADC. It is suggested that the therapeutic inhibition of Cyclin D1 protein may prove as a revolutionized therapeutic strategy for high-risk HPV infected cervical ADC patients. But further huge scale longitudinal studies implying larger numbers of cervical ADC patients are warranted to logistically validate and quantify the clinical significance of these proteins. Hence, briefly, this study may help in understanding the molecular mechanism modulating ADC of UC, designing the future studies, and treatment of these patients by targeting Cyclin D1 for improvement of global women’s health.

## Methods

### Tissue samples collection

The research and ethics committee of Safdarjang Hospital provided the approval before commencement of this study. 40 normal controls (UV-prolapse, non-neoplastic tissues) and 20 untreated ADCs with no family history of CC were enrolled from the Department of Obstetrics and Gynecology, Safdarjang Hospital, New Delhi, India. All the patients provided a written informed consent. Histopathological diagnosis along with the molecular analysis was done in all the samples by two independent pathologists. Also, the study confirms that all experiments were performed in accordance with relevant guidelines and regulations.

### Immunohistochemical Analysis

Fine sectioning (5 μm) was done in formalin-fixed paraffin-embedded specimens on poly-L-lysine (Sigma, St. Louis, MO, USA) coated slides, followed by immunohistochemistry (IHC). The antigen retrieval for Cyclin D1 was performed in 10 mM tris-EDTA buffer (pH-9.0) at 900W for 15 min/360W for 5 min. The sections were consecutively washed in Tris buffer saline (TBS; 0.1M; pH: 7.4) and then incubated with primary antibody of Primary rabbit monoclonal of Cyclin D1 (monoclonal, M3635, Dako cytomation Glostrup, Denmark; 1:200 dilution) overnight followed by the use of polymer based secondary antibody (Envision System peroxidase kit, DAKO, Carpinteria, CA) for 1 hr at room temperature after washing with TBS. Previous studies from our laboratory have mentioned the complete methodological details^9,10^.

### Scoring of IHC

The detailed scoring criterion for IHC results of Cyclin D1 protein was described by Tripathi *et al*.^9,10^ previously from our laboratory. Percentage positivity and intensity scores were added for calculating a total score value.

### Immunoblotting

Like previous studies from our laboratory, immunoblotting from the cervical tissues was done^9,10^. Polyclonal anti-human antibodies of Cyclin D1, β-actin (rabbit monoclonal β-actin antibody (1:2000, Abcam) and diluted secondary antibody HRP-conjugated rabbit anti-IgG (Abcam,) were used to determine the expression levels of Cyclin D1 in tumor tissues. Expression levels were further quantitated and compared in normal tissues and were evaluated by densitometry using Alpha Digidoc version 4.1.0 (Alpha Innotech Corporation, IL) as described in our previous reports^9,10^.

### Statistical Analysis

Statistical analysis was carried out using statistical software SPSS 20.0. Chi-square test was done to evaluate the expression of the Cyclin D1 protein in ADC. For overall comparison of Cyclin D1, JAG1, and Notch-3, non-parametric Kruskal Wallis test was used. Pairwise comparison was done through Mann Whitney’s U Test between groups (i) Cyclin D1 and JAG1, (ii) Cyclin D1 and Notch-3. p ≤ 0.05 is considered to be significant.

### Research data Statement

All data generated or analyzed during this study are included in this published article and its supplementary information files. The raw data files used in the article can be provided upon request.

## Supplementary information

Table S1, Table S2, Original blot

## References

[1] Ferlay J (2015). Cancer incidence and mortality worldwide: sources, methods and major patterns in GLOBOCAN 2012. Int J Cancer.

[2] Ganguly N, Parihar SP (2009). Human papillomavirus E6 and E7 oncoproteins as risk factors for tumorigenesis. J Biosci.

[3] Doorbar J (2012). The biology and life-cycle of human papillomaviruses. Vaccine.

[4] Halim TA, Farooqi AA, Zaman F (2013). Nip the HPV encoded evil in the cancer bud: HPV reshapes TRAILs and signaling landscapes. Cancer Cell Int.

[5] Das BC, Hussain S, Nasare V, Bharadwaj M (2008). Prospects and prejudices of human papillomavirus vaccines in India. Vaccine.

[6] Takeuchi S (2016). Biology and treatment of cervical adenocarcinoma. Chin J Cancer Res.

[7] Venkatesh V (2018). Targeting Notch signalling pathway of cancer stem cells. Stem Cell Investig.

[8] Choi K (2009). Distinct biological roles for the notch ligands Jagged-1 and Jagged-2. J Biol Chem.

[9] Tripathi R (2018). Jagged-1 induced molecular alterations in HPV associated invasive squamous cell and adenocarcinoma of the human uterine cervix. Sci Rep.

[10] Tripathi R (2014). Clinical impact of de-regulated Notch-1 and Notch-3 in the development and progression of HPV-associated different histological subtypes of precancerous and cancerous lesions of human uterine cervix. PLoS One.

[11] Ramdass B (2007). Coexpression of Notch1 and NF-kappaB signaling pathway components in human cervical cancer progression. Gynecol Oncol.

[12] Tripathi, R. *et al*. HES1 Protein Modulates Human Papillomavirus-Mediated Carcinoma of the Uterine Cervix. *J Glob Oncol*, 1–10 (2019). 10.1200/JGO.18.00141 PMC6426524 30615540

[13] Marampon F (2016). Cyclin D1 silencing suppresses tumorigenicity, impairs DNA double strand break repair and thus radiosensitizes androgen-independent prostate cancer cells to DNA damage. Oncotarget.

[14] Cho NH, Kim YT, Kim JW (1997). Correlation between G1 cyclins and HPV in the uterine cervix. Int J Gynecol Pathol.

[15] Balan R, Caruntu ID, Amalinei C (2013). The immunohistochemical assessment of HPV related adenocarcinoma: pathologic and clinical prognostic significance. Curr Pharm Des.

[16] Neyaz A (2017). Synchronous Cervical Minimal Deviation Adenocarcinoma, Gastric Type Adenocarcinoma and Lobular Endocervical Glandular Hyperplasia Along with STIL in Peutz-Jeghers Syndrome: Eliciting Oncogenesis. Pathways. Turk Patoloji Derg.

[17] Park H, Lee M, Kim DW, Hong SY, Lee H (2016). Glycogen synthase kinase 3beta and cyclin D1 expression in cervical carcinogenesis. Obstet Gynecol Sci.

[18] Tong R, Yang Q, Wang C, Bi F, Jiang B (2017). OVCA1 expression and its correlation with the expression levels of cyclin D1 and p16 in cervical cancer and intraepithelial neoplasia. Oncol Lett.

[19] Little L, Stewart CJ (2010). Cyclin D1 immunoreactivity in normal endocervix and diagnostic value in reactive and neoplastic endocervical lesions. Mod Pathol.

[20] Maliekal TT, Bajaj J, Giri V, Subramanyam D, Krishna S (2008). The role of Notch signaling in human cervical cancer: implications for solid tumors. Oncogene.

[21] Shin HM (2006). Notch1 augments NF-kappaB activity by facilitating its nuclear retention. EMBO J.

[22] Myong NH (2017). Altered expressions of Notch-1 signaling proteins and beta-catenin in progression of carcinoma *in situ* into squamous carcinoma of uterine cervix. Indian J Pathol Microbiol.

[23] Ronchini C, Capobianco AJ (2001). Induction of cyclin D1 transcription and CDK2 activity by Notch(ic): implication for cell cycle disruption in transformation by Notch(ic). Mol Cell Biol.

[24] Yao J, Duan L, Fan M, Yuan J, Wu X (2007). Notch1 induces cell cycle arrest and apoptosis in human cervical cancer cells: involvement of nuclear factor kappa B inhibition. Int J Gynecol Cancer.

[25] Mishra R, Nagini S, Rana A (2015). Expression and inactivation of glycogen synthase kinase 3 alpha/beta and their association with the expression of cyclin D1 and p53 in oral squamous cell carcinoma progression. Mol Cancer.

[26] Lu S, Zhang B, Wang Z (2005). Expression of survivin, cyclinD1, p21(WAF1), caspase-3 in cervical cancer and its relation with prognosis. J Huazhong Univ Sci Technolog Med Sci.

[27] Liang S (2013). CyclinD1, a prominent prognostic marker for endometrial diseases. Diagn Pathol.

[28] Bonilla C (2011). Cyclin D1 rare variants in UK multiple adenoma and early-onset colorectal cancer patients. J Hum Genet.

[29] Hayakawa Y (2011). Apoptosis signal-regulating kinase 1 and cyclin D1 compose a positive feedback loop contributing to tumor growth in gastric cancer. Proc Natl Acad Sci USA.

[30] Skomedal H, Kristensen GB, Lie AK, Holm R (1999). Aberrant expression of the cell cycle associated proteins TP53, MDM2, p21, p27, cdk4, cyclin D1, RB, and EGFR in cervical carcinomas. Gynecol Oncol.

[31] Bae DS (2001). Aberrant expression of cyclin D1 is associated with poor prognosis in early stage cervical cancer of the uterus. Gynecol Oncol.

[32] Schorge JO (2004). P16 as a molecular biomarker of cervical adenocarcinoma. Am J Obstet Gynecol.

[33] Koay MH, Crook M, Stewart CJ (2012). Cyclin D1, E-cadherin and beta-catenin expression in FIGO Stage IA cervical squamous carcinoma: diagnostic value and evidence for epithelial-mesenchymal transition. Histopathology.

[34] Casimiro MC, Crosariol M, Loro E, Li Z, Pestell RG (2012). Cyclins and cell cycle control in cancer and disease. Genes Cancer.

[35] Li Z, Wang C, Prendergast GC, Pestell RG (2006). Cyclin D1 functions in cell migration. Cell Cycle.

