# Author Correction: Cyclin D1 protein affecting global women’s health by regulating HPV mediated adenocarcinoma of the uterine cervix

**DOI:** 10.1038/s41598-020-63943-3

**Published:** 2020-04-29

**Authors:** Richa Tripathi, Gayatri Rath, Poonam Jawanjal, Mausumi Bharadwaj, Ravi Mehrotra

**Affiliations:** 1 Division of Molecular Genetics & Biochemistry, ICMR-National Institute of Cancer Prevention and Research (NICPR), Noida, India; 2 Division of Preventive Oncology, ICMR-National Institute of Cancer Prevention and Research (NICPR), Noida, India; 3 Department of Anatomy, VMMC & Safdarjung Hospital, New Delhi, India

Correction to: *Scientific Reports* 10.1038/s41598-019-41394-9, published online 22 March 2019

The original version of this Article contained errors.

The title included the “≤” symbol at the beginning as a result of typesetting error.

In addition, as a result of figure assembly errors, panels B and F of Figure 2 were duplicated from author’s previous research. This figure is now correct, and the raw data underlying it is now also included in the Supplementary Information file.

These have now been corrected in the HTML and PDF versions of this Article, and in the accompanying Supplemental Material.

