# Supplementary material for: ≤ Cyclin D1 protein affecting global women’s health by regulating HPV mediated adenocarcinoma of the uterine cervix
Source: Sci Rep. 2019 Mar 22;9:5019. doi: 10.1038/s41598-019-41394-9 (PMC6430791; doi:10.1038/s41598-019-41394-9)
Supplement: Supplementary file 1 — Table S1, Table S2, Original blot [file 41598_2019_41394_MOESM1_ESM.pdf]

Cyclin D1 protein affecting global women's health by regulating HPV mediated adenocarcinoma of the uterine cervix

Richa Tripathi, PhD[1,2]; Gayatri Rath, MS[3]; Poonam Jawanjal[3], PhD; Mausumi Bharadwaj, PhD[1]*; Ravi Mehrotra, MD[2]*

[1]Division of Molecular Genetics & Biochemistry, ICMR-National Institute of Cancer Prevention and Research (NICPR), Noida, India.

[2]Division of Preventive Oncology, ICMR-National Institute of Cancer Prevention and Research (NICPR), Noida, India.

[3]Department of Anatomy, VMMC & Safdarjung Hospital, New Delhi, India.

Supplementary Tables

Table S1. Test Performance of Cyclin D1 (nuclear) protein in ADC.

**Parameters (ADC)**	**Cyclin D1**
**Cut-off value (Total score)**	2.0
**Sensitivity (%)**	80
**Specificity (%)**	80
**AUC**	0.869
**p-value**	***0.0001**

Abbreviations: ADC, adenocarcinoma; AUC: Area under curve

*p-value ≤ 0.05 is significant

Table S2. Pairwise comparison between Cyclin D1 and Notch-3, Cyclin D1 and JAG1, Notch-3 and JAG1

Proteins	Mean Rank	Sum of Rank	p-value	MannWhitney U test	Wilcoxan test (W)
**HES1**<br>**Notch-3**	16.05<br>24.95	321.0<br>499.0	***0.015**	111.0	321.0
**HES1**<br>**Jagged-1**	26.33<br>14.68	526.0<br>293.0	***0.0001**	83.5	293.5
**Notch-3**<br>**Jagged-1**	28.48<br>12.53	569.5<br>250.5	***0.0001**	40.5	250.5

*p-value ≤ 0.05 is significant

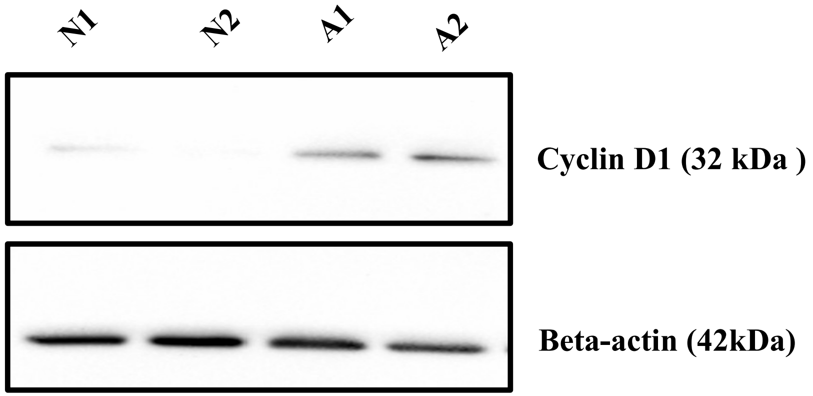

Figure 2f

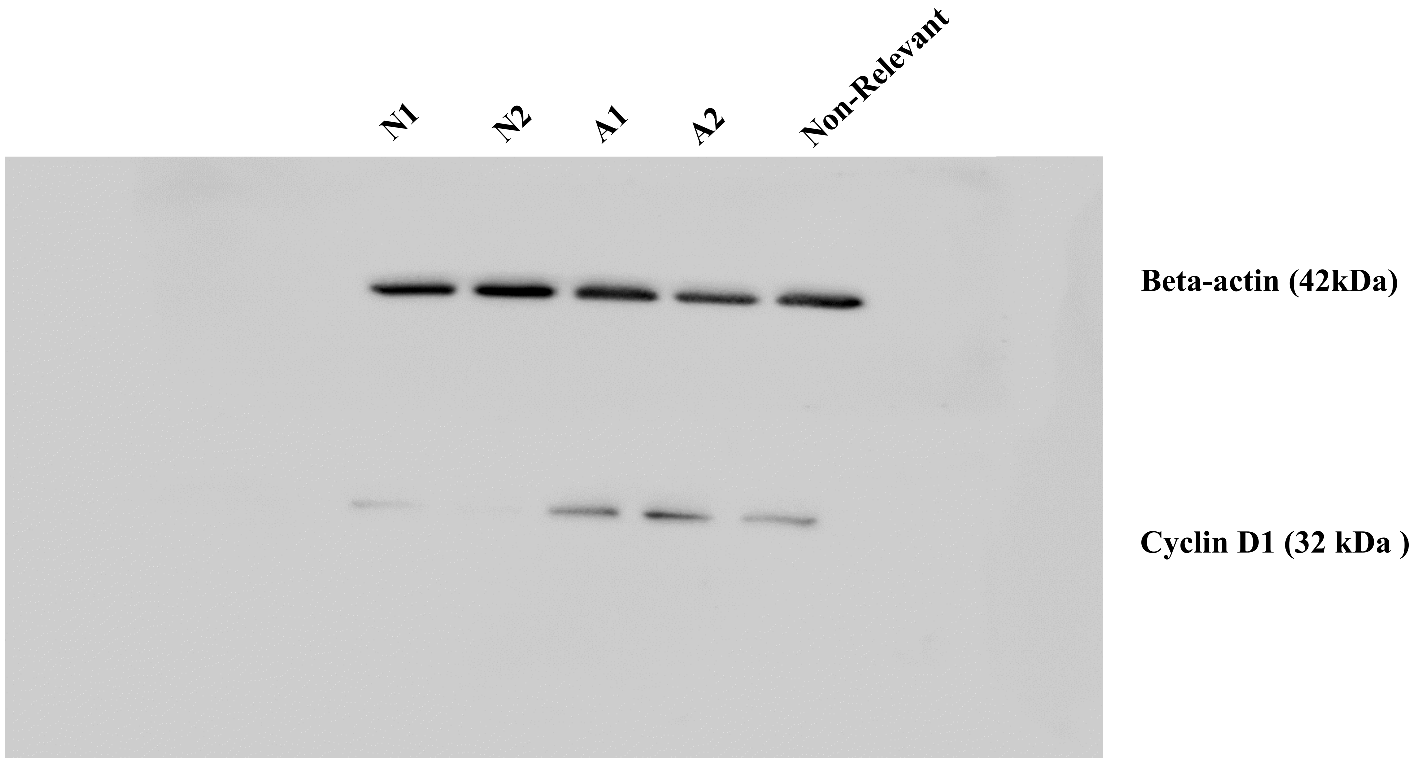

Figure 2f (Original blot)
